# Supplementary material for: “…I feel like I am just staying here waiting for death”: A qualitative study of the lived experiences of people with advanced illness in refugee settlements in Uganda
Source: PLOS Glob Public Health. 2025 Dec 29;5(12):e0005541. doi: 10.1371/journal.pgph.0005541 (PMC12747433; doi:10.1371/journal.pgph.0005541)
Supplement: S1 Fig — Base map data © OpenStreetMap contributors (ODbL). Map annotations by the authors. (DOCX) [file pgph.0005541.s001.docx]

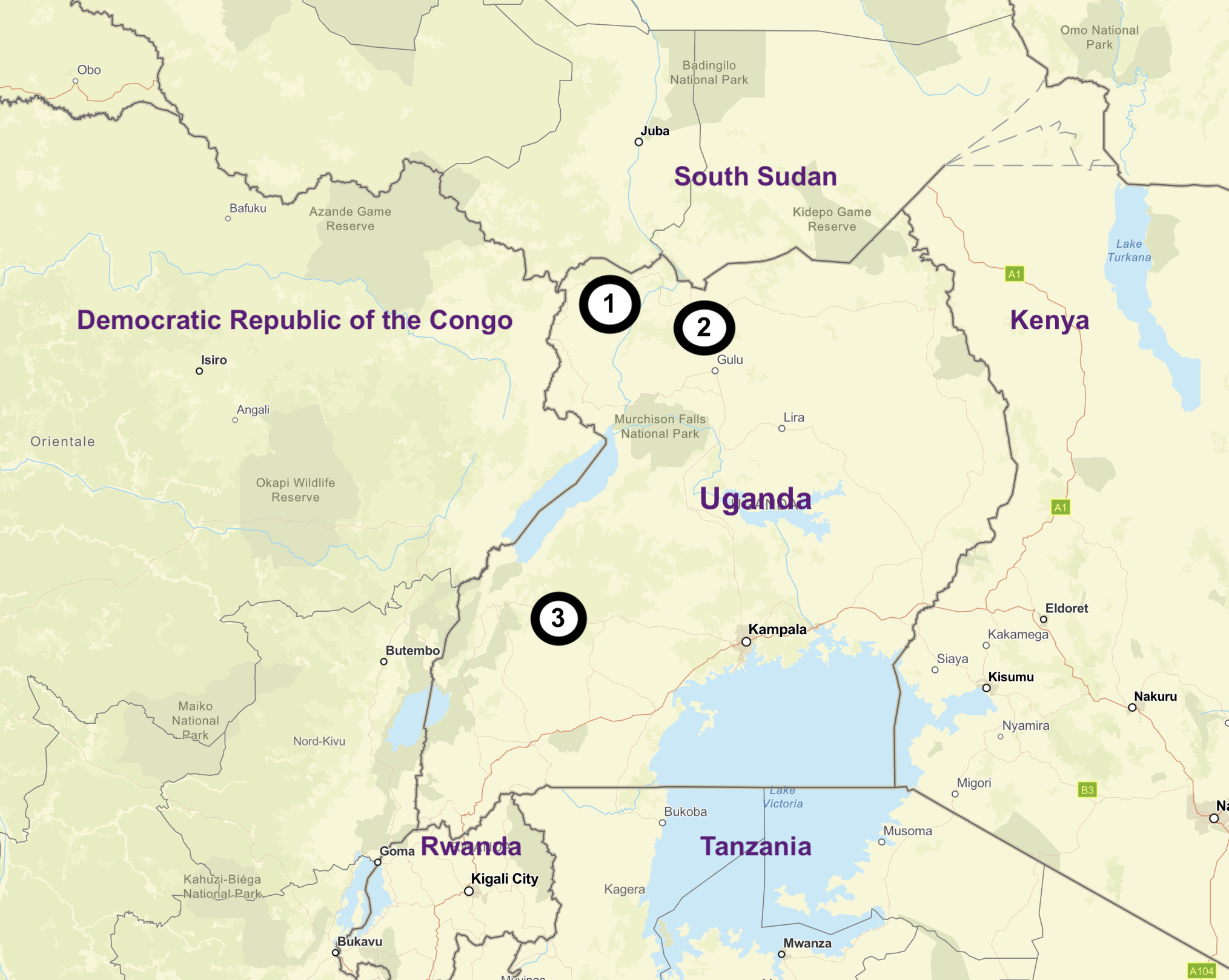


**Key:**

Bidibidi Refugee Settlement

Nyumanzi Refugee Settlement

Kyaka II Refugee Settlement

**S1 Fig: Location of Study Sites.** Base map data © OpenStreetMap contributors (ODbL). Map annotations by the authors.
