# Supplementary material for: “…I feel like I am just staying here waiting for death”: A qualitative study of the lived experiences of people with advanced illness in refugee settlements in Uganda
Source: PLOS Glob Public Health. 2025 Dec 29;5(12):e0005541. doi: 10.1371/journal.pgph.0005541 (PMC12747433; doi:10.1371/journal.pgph.0005541)
Supplement: Attachment — Submitted filename: PGPH-D-25-02762_reviewer_comments_2025_12_02.pdf [file pgph.0005541.s003.pdf]

PGPH-D-25-02762_reviewer_comments

Overall impression of the revised manuscript

The methodology was reviewed and consolidated, addressing the calculation errors, the data collection risks, and the potential coding bias.

In addition, the manuscript was integrated into a larger, technical (medical/health system), operational (3D nexus), and political (national authorities) discussion, alerting the international community on underrepresented patients in fragile settings.

I thank the authors for integrating a tighter theoretical framework, and a deeper analytical section. This research will surely contribute to, improving field operations, and ultimately patients' care.
I recommend the manuscript for publication.

Confidential elements to the editors

- Thank you for your reviewing time, asking clarifications on the 17 authors' roles. Despite, the details on their respective responsibilities, I still think it is excessive. That being said, it is not an obstacle for a publication.
