# Supplementary material for: “…I feel like I am just staying here waiting for death”: A qualitative study of the lived experiences of people with advanced illness in refugee settlements in Uganda
Source: PLOS Glob Public Health. 2025 Dec 29;5(12):e0005541. doi: 10.1371/journal.pgph.0005541 (PMC12747433; doi:10.1371/journal.pgph.0005541)
Supplement: Attachment — Submitted filename: Response to reviewers.docx [file pgph.0005541.s002.docx]

| **Reviewer comment** | **Author response** |
| --- | --- |
| **Editor** | |
| Can you please explain why there are 17 author on a manuscript with 44 participants? Please provide a detailed description of what every author has contributed and please ensure that you review IJME author guidelines. | Thank you for your query. We confirm that all authors meet the ICMJE authorship criteria, and we provide a clear statement of contributions in accordance with the journal requirements. The study was conducted across three refugee settlements, involving multiple languages and local cultural contexts, and required work across community engagement, ethics approvals, site-level access negotiation, multilingual interviewing, translation validation, and cross-site thematic interpretation. For this reason, the research team necessarily included local clinical and community-based practitioners, interviewers fluent in relevant languages, and academics with expertise in palliative care, refugee health, humanitarian response, and qualitative research. In addition, the interpretation and policy implications of the study required input from experts in global palliative care system development and health policy, to ensure that recommendations were grounded in current WHO, Sphere, and humanitarian coordination frameworks. These contributors played a substantive role in conceptual framing, interpretation of findings, and translation of results into actionable practice and policy recommendations. |
| Please include a complete copy of PLOS’ questionnaire on inclusivity in global research in your revised manuscript. Our policy for research in this area aims to improve transparency in the reporting of research performed outside of researchers’ own country or community. The policy applies to researchers who have travelled to a different country to conduct research, research with Indigenous populations or their lands, and research on cultural artefacts. The questionnaire can also be requested at the journal’s discretion for any other submissions, even if these conditions are not met. Please upload a completed version of your questionnaire as Supporting Information when you resubmit your manuscript. | We have included a copy of the completed questionnaire with our submission. |
| **Reviewer 1** | |
| The article is looking into refugees’ access to health services, zooming on individuals with severe illnesses, in need of palliative care. The authors analyze the status of those people’s access to health services, through the lens of their experience living in camps in Uganda. The article argues that patients with advanced illnesses face structural violence from political and social systems, which syndemic dynamic accentuates preventable suffering.  Considering the points of discussion detailed below, I suggest for the authors to review their arguments and calculations before re-submitting the article to PLOS Global Public Health, to be considered for a second round of review. Even though the case study of palliative care, isn’t discussed enough in the context of a refugee camps, I recommend a deeper review of the theoretical frameworks on the 3D Nexus and Camp Management, as well as a stronger argument towards patients with advanced illnesses. This will strengthen the article on the current gap in the literature and the exploration of paths, building a response to palliative care in fragile contexts.  My initial point concerns the services under the umbrella of palliative care. Given the severity of the patients’ health conditions at such medical stage, a safe and efficient pharmaceutical follow up as well as regular consultations, are of outmost importance. In that regard, a fragmentation of services and their frequency, could critically impair patients’ conditions, and quickly escalate the degradation of their health. If this conclusion is detailed in the article, I suggest to make a **tighter link between the interviewees’ health perception, and the actual quality of care they are receiving – describing the type and frequency of the services they have had inside and outside the camp**. | We thank you for your thoughtful and constructive suggestions. In response to your feedback, we have made the following focused revisions to strengthen conceptual framing, analytic clarity, and the description of the contexts of care provided across the three settlements:  First, to provide further detail around the availability of services and care available for interviewees, we added a paragraph to the Setting section that outlines the organisation and typical frequency of care for people with advanced illness across the settlements (lines 152 – 159). For example, we now describe how:  “*Primary facilities provide holistic care, including symptom management, but have limited diagnostic capacity and intermittent availability of essential medicines, including opioids. … Palliative care teams visited settlements to provide counselling, oral morphine where available, and home-based support, but the regularity of visits differed between sites due to staffing, caseload, and logistical constraints.”*  Second, in the Results section we strengthened the analytic link between participants’ reports of worsening health and the continuity and coordination of care. In Theme 3 and Theme 4 we now explicitly draw out and reflect how participants themselves connected their physical deterioration to irregular review, difficulty securing medications, and fragmented referral pathways (lines 590 – 594) and lines 678 – 685). For example, we added:  “*Across accounts, participants’ perceptions of worsening health were closely linked to the fragmented and intermittent nature of care… This discontinuity contributed to a sense that deterioration was both expected and unavoidable*.”  This has not involved added new findings or data, but instead making the interpretation clearer to better link health perception and care quality.  Third, to refine the manuscript to reflect deeper engagement with the Humanitarian–Development–Peace (3D) Nexus and Camp Management frameworks, we have added content to the Discussion. We now explicitly use the 3D Nexus to interpret how care fragmentation arises in protracted displacement settings and why this is particularly significant for palliative care. We highlight that palliative care needs in refugee settlements are not always short-term or confined to the very end of life; rather, individuals may require sustained, adaptive support across fluctuating periods of stability and deterioration (lines 731– 737). For example, we note:  “*Strengthening palliative care in settlements therefore requires coordinated approaches that operate across systems and over time, recognising that many people with advanced illness will require support that is sustained, adaptive, and embedded within routine care provision. This aligns with evidence from palliative care programmes in Uganda and neighbouring countries, where longitudinal symptom management, psychosocial support, and home- and community-based care models have been shown to be feasible and effective (29, 30).”*  Finally, we clarified the purpose and contribution of Table 3, explicitly linking it to participants’ experiences and positioning it as offering context-sensitive, feasible response options (lines 862 – 866). We added:  “*Table 3 provides a practical synthesis that maps the needs identified by participants to actionable responses and resource requirements. This offers a feasible route to strengthening palliative care in refugee settlements without establishing new or parallel systems by improving coordination between existing humanitarian and national health system structures.*” |
| A second point of attention concerns the aim of humanitarian actors with regards to health services operating in fragile contexts. Initially, a refugee camp is a situation which responds to an emergency, not a planned system agreed upon. In that context, the initial objective aims at addressing the emergencies which abide by the method of triage. People are seen by medical providers, according to the patients’ flow of the moment and the urgency of their health conditions. As a result, the “severe illnesses” criteria could be weighted down in front of, more severe cases. As such, I suggest to make a **stronger argument to how triage affects health access to patients with severe illnesses, and how this differs from the structural violence that all refugees face**. | We thank the reviewer for raising this important point regarding the origins and aims of humanitarian health provision and how triage-based emergency logics shape access to care in refugee settlements. We agree that palliative care needs can be deprioritised—not because they are less severe, but because the system remains structured around a crisis-response logic.  To address this, we have revised the Discussion to explicitly articulate the distinction between emergency triage as an appropriate response in the acute phase of displacement, and ongoing care discontinuities that arise when emergency-oriented systems are not reconfigured for long-term, progressive conditions (lines 717 – 726):  *“In many settlement health systems, service delivery remains structured around emergency-oriented triage established during the acute phase of displacement, where prioritisation is based on immediacy and threat to life. As displacement becomes protracted, the continued application of these emergency models results in systematic under-recognition of chronic and advanced illness needs, particularly given the rising burden of non-communicable disease and multi-morbidity among displaced populations. This represents a distinct form of institutional exclusion, separate from broader structural violence, that emerges when systems built for rapid crisis response are not reconfigured for long-term care* *or with health system resilience in mind.”*  We sought to align this with your request to frame discussions within the Humanitarian–Development–Peace (3D) Nexus, we highlight that the strengthening of palliative care in settlements requires coordinated approaches that operate across systems and over time, recognising that many people with advanced illness will require support that is sustained, relational, and embedded within routine health system functioning. |
| A final impression touches upon the status of a refugee camp. As mentioned, camps aren’t structured and managed with a definite settlement perspective in mind. Advocacy campaigns targeting local authorities, are often carried out to integrate refugees to the host communities via, for example, public health policies. However, settlements are too often and for a long-period of time, operating “off the grid” by the international community, creating a parallel health system. The unfortunate downside of emergency responses in camps, is that they last too long, and fragment of the continuum of care. That being said, there is a great amount of literature on the limit to what humanitarian operators can do within the boundaries of a refugee camp, and the bridges they build with the host country health system. I suggest a **deeper focus on the 3D nexus, and the strategy within which aid actors (humanitarians, stabilization, and development) are currently operating**. | We thank the reviewer for highlighting the importance of linking our findings to the organisation of health services in refugee settlements. We agree that settlements are commonly structured around an emergency response model, and that this approach often persists long after displacement becomes protracted, and how this can result in parallel systems with limited integration into national health planning, which has particular consequences for people living with advanced illness whose needs require ongoing and coordinated care.  To reflect this, and as highlighted in response to an earlier comment, we have revised the Discussion to more clearly centre the Humanitarian–Development–Peace (HDP/3D) Nexus as a conceptual frame guiding our interpretation of fragmentation. We now explicitly describe how emergency-oriented triage logics can become structurally embedded when displacement remains unresolved, and how this leads to a form of institutional exclusion specific to chronic and advanced illness. We also clarify that strengthening palliative care requires shifting from short-term humanitarian provision toward coordinated and relational care embedded within ongoing health system functioning, rather than parallel or temporary service structures (lines 709 – 732):  *“The Humanitarian–Development–Peace (HDP/3D) Nexus offers a lens for understanding these patterns, emphasising the alignment of immediate humanitarian relief with longer-term health system strengthening and governance in protracted crises. … In many settlement health systems, however, service delivery remains structured around emergency-oriented triage established during the acute phase of displacement… … Strengthening palliative care in these settings therefore requires coordinated approaches that operate across systems and over time.”* |
| There are 4 major issues I recommend to review:  An element to be explained is the timing between the different data collection periods. Line 196 to 199: the first set is between July and November 2022, then in April 2024 – almost a year and 5 months after, then between August and November 2024.   - Considering the 2022-2024 gap, how did you stabilize external factors which could have biased your study? (Like a change in Public Health Policies, Change in international fundings, etc....) | We thank the reviewer for this helpful observation. The study was designed as a multi-site, phased qualitative study, in which data collection proceeded in stages due to (1) ethical approvals required across administrative districts, (2) negotiated access with implementing partners, and (3) the practical realities of conducting research in humanitarian settings. We recognise that the time gap between the 2022 and 2024 data collection periods could raise questions regarding changes in contextual conditions.  To address this, we have clarified the rationale for staggered data collection and described how we accounted for contextual continuity in the manuscript. Specifically, we have now revised the Data Collection section of the Methods to note that (lines 218 – 226):  *This phased approach reflected the time required to obtain site-specific ethical approvals and negotiate access agreements with implementing partners across each settlement. To ensure consistency across data collection phases, the same interview guides were used across all sites, interviewers were trained with a shared protocol, and reflexive field notes were maintained. During the period of data collection, there were no substantive changes to national refugee health policy, settlement health service organisation, or palliative care provision models, as confirmed through regular consultation with district health leads and partner NGOs.*  Furthermore, we have added a clarifying sentence in the Limitations section (lines 832 – 838):  *…data collection occurred in phased periods across 2022 and 2024. While this enabled the inclusion of multiple settlements, it may also have introduced contextual variation over time. However, national refugee health policy, settlement health system organisation, and palliative care outreach models remained stable during this period, and shared interview guides, training protocols, and reflexive field notes were used to support consistency across phases.* |
| An element I recommend to strengthen, concerns the interviewers and their influence on the type and quality of the data. Line 172 (health workers); line 199-201 (2 researchers); line 202 (clinical officer and a psychologist; then a social anthropologist and a social worker); line 205-2026 (2 nurses, then a nurse and a social worker).   - How did you train the data collectors to harmonize the data collection? | We thank the reviewer for highlighting this point. The study team recognised this risk and took deliberate steps to support consistency and rigour across interviewers which we have now reflected in the Data Collection section of the Methods (lines 233 – 241). We outline how, before data collection commenced at each site, all interviewers participated in a shared training programme that included i) a joint orientation to the study aims, research questions, and positionality considerations; ii) standardised training on the semi-structured interview guide, including role-play and iterative refinement; iii) guidance on trauma-informed interviewing, given the anticipated emotional sensitivity of the topic; iv) discussion of power dynamics and strategies for enabling participant-led narration; and iv) capturing field notes to document context, tone, interviewer influence, and post-interview reflection.  Training was delivered collaboratively by senior team members (AA, EN, MA) with clinical, qualitative research, and palliative care expertise. Interviewers also engaged in regular debriefing meetings via Zoom during fieldwork to reflect on emerging themes, clarify interpretations, and ensure alignment in interviewing approach.  We also added a clarifying sentence in the Analysis section (lines 279 – 281) to show how harmonisation continued into interpretation: “*Furthermore, regular cross-site reflexive discussions between interviewers and analysts supported interpretive consistency and helped mitigate the influence of interviewer backgrounds and the phased nature of data collection*.” |
| In addition, if the article briefly mentions the interviewers’ background as a limit, I recommend to be specific on how the authors addressed this, at different steps of the study: specifically, when the data were collected via the semi-structured interviews, and during the back and forth for the coding and analysis. For those 2 times, during which interviewers worked with coders to complete the translation (line 212):   - How did you control or factor-in, the influence of the interviewers on the coder and the analyst, especially considering the 5 languages of the study? - Did the independent authors spoke both the 5 languages to ensure the triangulation (line 221-225)? | Thank you for highlighting interviewer positionality, and we have expanded the reporting around data collection and data analysis to clarify our approach.  We detail in the Methods (Data Collection and Data Analysis sections), how study materials (including consent forms and interview guides) were translated into French, Swahili, Juba Arabic, Maʼdi, and Lugbara to ensure accessibility and comprehension across settlements (lines 250 – 252). We have expanded the Methods section to outline that interviews were conducted in the language preferred by each participant (lines 252 – 256). Across the three sites, interview languages included English, Juba Arabic, Swahili, Luganda, Kakwa, Bari, Lugbara, Keliko, and Dinka, reflecting the linguistic diversity of the settlements. Interviewers were fluent in the predominant local languages and culturally familiar, with a view to supporting rapport and reducing linguistic hierarchy during interviews.  To minimise the influence of interviewer perspective in transcription, translation, and interpretation, we used the following approach which has been further detailed in the Data Collection section of the Methods (lines 248 – 262):   - Interviewers who conducted the interviews completed a full transcription and translation of the interview into English. - A second bilingual team member reviewed translated transcripts to ensure the meaning of participants’ words, idioms, and expressions was preserved) alongside checking cultural nuance such as tone and implied meaning.   In the Data Analysis section (lines 273 – 281) we outline that:   - All coding was conducted in English to ensure a shared analytic dataset. - Interviewers were involved in team discussions during coding and theme development to clarify interpretations of idiomatic, emotional, and cultural meaning. - We also incorporated triangulation during analysis, with two researchers independently coding subsets of transcripts.   This approach enabled us to ensure that linguistic meaning was interpreted through discussion, without requiring that all coders speak every language represented in the dataset.  We have also added an acknowledgement in the Limitations section (lines 841 – 846) that there may have been some residual constraints to the interpretation of data resulting from the multiple languages and approach to analysis. |
| The calculation included in the Table 2 pages 13 and 14, needs to be reviewed.   - The percentage for the variables, sex, diagnosis, performance, education, marital status, caregiver presence, don’t seem to equal 100%. (For example: 27 women interviewed out of 44 participants is 61,3%, not 56,25% as mentioned line 247 and in the table) - Diagnosis – Cancer (unknown type): the only the data entered is “1”, in the table for Bidibidi but the total is recorded as 2. The total of participants for this variable becomes 45 instead of the initial 44. | Thank you very much for your comment. We have checked and amended the values relating to percentages, and revised the unknown cancer type data presented in Table 2. |
| There are 2 minor issues I recommend to review:  The initial point concerns the participants, for the pre-test and the study itself:   - Line 194, the number of participants should be precise. Is it 2 or 3? I suggest to be specific. I would also recommend adding information on how those 2 or 3 participants were identified to strengthen the methodology. | Thank you. The topic guide was piloted with three individuals living with advanced illness in Bidibidi Refugee Settlement. These individuals were identified by community health workers supporting palliative care outreach, based on their ability to participate comfortably in an interview and willingness to provide feedback on question clarity. The pilot interviews were used solely to refine the guide and were not included in the analysis. We have reflected this in the Data Collection section of the Methods. |
| The elements described in lines 743-745, are part of the essential health care package of services provided by local and international organizations. There are included in the CHS and Sphere standards, and should be a standard practice.   - I recommend to clarify the source of your argument. I also suggest adding a published reference and/or specific information on the context. | Thank you. We have expanded our text here to highlight our intention that was to emphasise that implementation remains inconsistent in practice, especially in protracted displacement settings. We recognise that palliative care is formally recognised within global humanitarian and health system guidance and have acknowledged this in the revised text whilst reflecting on what factors may need to be considered to support realisation in practice. Our revised text (lines 850 – 859) now reads as follows:  *Whilst palliative care is recognised within global humanitarian health standards (e.g., the Sphere Handbook (36) and WHO guidance on palliative care integration in humanitarian emergencies and crises(62)), implementation remains limited and uneven. Realising these standards in practice requires reliable access to essential medicines (including opioids for pain relief), training and mentorship for health workers at multiple levels, integration of palliative care into routine primary care and referral pathways, and coordination across humanitarian and national health systems. These efforts should be grounded in contextually meaningful psychosocial, spiritual, and community-engaged approaches(62).* |
| **Reviewer 2** | |
| Thank you for the opportunity to read this manuscript, which examines the experiences of (refugees) people living with advanced illness in Uganda. The study is both important and timely. In many conflict-affected countries, populations are ageing and the incidence of advanced disease is increasing alongside rising life expectancy. As a result, this issue will become an even more critical theme in the coming years, particularly in humanitarian and low-resource settings where health systems are already overstretched. The article is carefully executed and offers significant added value to the field of global health research, as it highlights multiple barriers and traumatic experiences faced by individuals in need of palliative care, as well as those living as refugees.  The research question is clearly expressed and well justified. The chosen framework (a phenomenological paradigm) is highly appropriate for a study that seeks to explore personal experiences and subjective meanings of lived reality. The thematic analysis is well structured and thoughtfully divided into suitable subthemes. The interviews successfully bring out the authentic voices of the participants. This indicates a strong level of trust between interviewers and respondents, as the material includes highly sensitive, painful, and even taboo experiences that individuals would not easily disclose without a sense of safety and rapport. The results are presented clearly and coherently. | We thank the reviewer for their positive and encouraging feedback. We appreciate the recognition of the study’s importance, methodological appropriateness, and the clarity of the findings, and we are pleased that the sensitivity and depth of participant narratives were evident. |
| Minor suggestions 1) The reader might benefit from a simple map of Uganda indicating the locations of the settlements where interviews were conducted, as this would provide contextual understanding of the study sites | Thank you for this suggestion. We have created a map and included it as a supplementary file (Supplementary File 1). We have added a sentence directing readers to the map in Table 1 where the geographical locations of the settlements are outlined. |
| 2) The ethics statement is written twice in the manuscript (lines 120-122 and 238-241) Remove duplication | Thank you. We have retained only the text at the beginning of the Methods section, under a subheading 'Ethics Statement', aligned with a request by the journal for placement of the statement. We deleted the repeated text at the end of the Methods section. |
| I recommend the manuscript for publication with minor revisions. This important work will make a valuable contribution to the literature on palliative care in humanitarian settings. Thank you. | Thank you very much. |
